# Clustering of lifestyle behaviors and adiposity in early adolescents in Spain: findings from the SI! Program for Secondary Schools

**DOI:** 10.1186/s12889-023-16461-6

**Published:** 2023-08-12

**Authors:** Patricia Bodega, Gloria Santos-Beneit, Amaya de Cos-Gandoy, Luis A. Moreno, Mercedes de Miguel, Xavier Orrit, Anna Tresserra-Rimbau, Jesús Martínez-Gómez, Sonia L. Ramírez-Garza, Emily P. Laveriano-Santos, Camila Arancibia-Riveros, Ramón Estruch, Rosa M. Lamuela-Raventós, Rodrigo Fernández-Jiménez, Juan M. Fernández-Alvira

**Affiliations:** 1 Foundation for Science, Health, and Education (SHE), 08011 Barcelona, Spain; 2 Centro Nacional de Investigaciones Cardiovasculares (CNIC), 28029 Madrid, Spain; 3 Icahn School of Medicine at Mount Sinai, The Zena and Michael A. Wiener Cardiovascular Institute, New York, NY 10029 USA; 4 GENUD (Growth, Exercise, NUtrition and Development) Research Group, Faculty of Health Sciences, Instituto Agroalimentario de Aragón (IA2), University of Zaragoza, and Instituto Investigación Sanitaria de Aragón (IIS Aragón), 50009 Zaragoza, Spain; 5 Fisiopatología de La Obesidad Y Nutrición (CIBERObn), Consorcio CIBER, Instituto de Salud Carlos III (ISCIII), 28029 Madrid, Spain; 6 Department of Nutrition, Food Science and Gastronomy, School of Pharmacy and Food Sciences XIA, Institute of Nutrition and Food Safety (INSA‐UB), University of Barcelona, 08921 Santa Coloma de Gramenet, Spain; 7 Department of Internal Medicine, Institut d’Investigacions Biomèdiques August Pi I Sunyer (IDIBAPS), Hospital Clínic, University of Barcelona, 08036 Barcelona, Spain; 8 Hospital Universitario Clínico San Carlos, 28040 Madrid, Spain; 9 Centro de Investigación Biomédica En Red en Enfermedades CardioVasculares (CIBERCV), 28029 Madrid, Spain

**Keywords:** Teenagers, Adiposity, Lifestyle pattern, Cluster analysis, Childhood obesity

## Abstract

**Background:**

Several unhealthy lifestyle behaviors in adolescence are often linked to overweight/obesity. Some of them may be present simultaneously, leading to combined effects on health. Therefore, the clustering of several unhealthy behaviors in adolescents might be associated with adiposity excess.

**Purpose:**

To identify lifestyle patterns and analyze their association with adiposity in early adolescents.

**Methods:**

A cross-sectional cluster analysis was performed in 1183 adolescents (50.5% girls) with a mean age of 12.5 (0.4) years included in the SI! Program for Secondary Schools in Spain to identify lifestyle patterns based on healthy diet, step counts, sleep time, and leisure screen time. Generalized mixed models were applied to estimate the association between lifestyle patterns and adiposity indices.

**Results:**

Four lifestyle patterns were derived: *Cluster 1-higher screen time and poorer diet* (*n* = 213), *Cluster 2-lower activity and longer sleepers* (*n* = 388), *Cluster 3-active and shorter sleepers* (*n* = 280), and *Cluster 4*-*healthiest* (*n* = 302). Except for the number of steps (12,008 (2357) day), the lifestyle behaviors in our sample presented levels far below the recommendations, especially for sleep duration. *Cluster 4* included the largest proportion of adolescents from high socioeconomic status families (47.7%) and the lowest prevalence of overweight/obesity (23.1%). Compared to *Cluster 4*-*healthiest*, adolescents in the remaining clusters presented a higher prevalence of overweight/obesity and central obesity, showing *Cluster 3* the highest prevalences (PR:1.31 [95%CI: 1.31, 1.31] and PR:1.40 [95%CI: 1.33, 1.47]).

**Conclusions:**

Clustering of lifestyle patterns in early adolescence allows the identification of individuals with excess adiposity, in whom health promotion strategies should be stressed, especially in socioeconomically disadvantaged groups.

**Trial registration:**

Clinical Trial Registry, NCT03504059. Registered 20/04/2018—Retrospectively registered, https://clinicaltrials.gov/ct2/show/NCT03504059.

**Supplementary Information:**

The online version contains supplementary material available at 10.1186/s12889-023-16461-6.

## Introduction

According to the World Health Organization, the prevalence of overweight and obesity among children and adolescents has risen dramatically from just 4% to just over 18% [1]. Strong evidence suggests that childhood obesity is a risk to develop chronic diseases during adulthood like type 2 diabetes, cardiovascular (CV) diseases, cancer, and mental health disorders [2, 3]. Regarding specific lifestyle behaviors, there is established evidence in the literature showing that a sedentary lifestyle and high screen time are associated with unhealthier anthropometric indicators [4, 5], with an increased risk of overweight/obesity and their continuation into adulthood [6–8]. Sleep time and sleep quality also play an important role in obesity development [9–12]. Lifestyle habits are interrelated and linked to adiposity levels; it is well known that improving one component positively influences the rest [7, 10, 13]. Assessing the clustering of several behaviors considers the existence of multiple and interactive influences on lifestyles. This approach can facilitate understanding which behaviors are often combined and linked to obesity, and in which subgroups of the population prevention initiatives are most needed. The aim of this study was to identify and analyze the relationship between lifestyle patterns and adiposity in early adolescents included in the SI! Program for Secondary Schools (*Salud Integral* – Comprehensive Health) trial.

## Materials and methods

### Study design and sample

The SI! Program for Secondary Schools is a cluster-randomized controlled intervention trial (NCT03504059. Registered 20/04/2018—Retrospectively registered, https://clinicaltrials.gov/ct2/show/NCT03504059) involving 1326 adolescents attending public secondary schools in Barcelona and Madrid, Spain. The study was approved by the corresponding ethics committees, and all participants gave written informed consent. All details on recruitment and trial design have been published previously [14].

The present study included 1183 participants aged 11–14 years with complete baseline information (obtained in 2017) for the variables included in the lifestyle patterns analysis: diet, number of steps, sleep time, and leisure screen time.

### Dietary intake

Adolescents answered a validated 43-item food frequency questionnaire (Children’s Eating Habits Questionnaire) [15, 16] related to their food consumption during the preceding 4 weeks. The questionnaire was guided by trained nutritionists and completed by the adolescents via an online application. Response options were ‘Never/less than once/week’, ‘1–3 times/week’, ‘4–6 times/week’, ‘1 time/day’, ‘2 times/day’, ‘3 times/day’, ‘ ≥ 4 times/day’, and ‘I have no idea’ [16]. Participants with more than 50% of answers missing or who answered ‘I have no idea’ to all questions were excluded from the analysis. For the analysis, a conversion factor ranging from 0 (‘I have no idea’ or ‘missing data’) to 30 (≥ 4 times/day’) was used to transform answers into weekly consumption frequencies [17]. For missing data in water intake, the data was imputed according to mean intake by age [17].

Based on this questionnaire, three dietary patterns (*Processed*, *Traditional* and *Healthy*) were derived by principal component analysis using a protocol previously described [18]. The *Healthy diet* pattern was selected as an overall indicator for healthy diet in the lifestyle clusters. It was characterized by high loadings on vegetables, fruits, unsweetened cereals and dairy, non-fried meat and fish, dried fruits and whole bread, and low loadings on sauces, white bread, chocolate, sweets, snacks, and sweetened drinks.

### Step counts

Adolescents wore an accelerometer (Actigraph wGT3X-BT) on their non-dominant wrist for 7 consecutive days except when bathing or swimming. Step counts were estimated by the pedometers included in the devices. Records were considered valid if they provided data from a minimum of 4 days with at least 600 min per day of wear time [19].

### Sleep time

Sleep time was assessed by accelerometry (Actigraph wGT3X-BT) according to the cutoff points proposed by Cole-Kripke et al. [20–22]. Sleep records were considered valid if they provided data from a minimum of 4 nights of wear time with a maximum of 960 min of sleep per night.

### Screen time

Passive and active leisure screen time was assessed through two questions adapted from the Spanish National Health Survey [23]: 1) ‘*How much leisure time per day do you spend watching TV/series/videos?’* for passive screen time, and 2) ‘*How much leisure time per day do you spend playing with a computer/tablet/videogame…?’* for active screen time. Response options were ‘maximum 1 h/day’, ‘maximum 2 h/day’, ‘maximum 3 h/day’, or ‘ ≥ 3 h/day’, separately for weekdays and weekends. Daily screen hours were calculated as follows: *total screen time* = *((5* × *passive screen time on weekdays* + *2* × *passive screen time on weekends)/7)* + *((5* × *active screen time on weekday* + *2* × *active screen time on weekends)/7)*.

### Anthropometric measurements

All participants were instructed to fast overnight before measurements. Trained nutritionists measured participants’ weight and percentage of body fat through bioelectrical impedance analysis (OMRON BF511 body composition scale), height (Seca 213 stadiometer), and waist circumference (WC) (Holtain tape) [24]. Body mass index (BMI) was calculated as body weight divided by height squared (kg/m^2^). Fat mass was calculated by multiplying the percentage of body fat by weight (kg) and dividing by 100. Fat mass index (FMI) was calculated by dividing body fat mass by height squared (kg/m^2^). Waist-to-height ratio (WHtR) was calculated by dividing WC (cm) by height (cm). Age- and sex-adjusted z-scores were calculated according to the Center for Disease Control (CDC) standards for BMI [25]; and the Third National Health and Nutrition Examination Survey (NHANES III) cutoff points for WC and WHtR [26]. Specific age and sex FMI z-scores were calculated based on our sample. The participant age range was 11–14. Participants aged 13 and 14 (n = 88 and n = 12, respectively) were combined into a single age group (13 years) to calculate the age- and sex-specific z-scores of FMI. Maturation stage (from I to V) according to Tanner [27] was self-reported by participants with the support of pictograms. Missing values in anthropometric variables were not imputed.

### Socioeconomic status

Socioeconomic information was reported through the family questionnaire. Household income was categorized into three levels based on the average annual household income in Spain in 2016 (26,730 €), the most recent data available at the time of recruitment [28] (‘below average’, ‘average’, and ‘above average’). Parental education level was categorized into three levels according to the International Standard Classification of Education (ISCED) [29]: low (secondary studies or below, ISCED levels 0 to 3), medium (postsecondary non-tertiary education or short-cycle tertiary education, ISCED levels 4 to 5), and high (university studies, ISCED levels 6 to 8) taking as a reference the highest educational level reported in the household. Migrant background was assumed when at least one of the parents was born outside Spain. Socioeconomic status was classified as unknown if no information was available.

### Statistical methods

Cluster analysis was performed in order to construct groups or clusters ensuring that within a group the observations are as similar as possible, while observations belonging to different groups are as different as possible, based on four lifestyle variables: healthy diet, step counts, sleep time, and leisure screen time. Z-scores were calculated to standardize the variables and prevent some from having more weight than others in the final solution [18]. Lifestyle clusters were derived through a combination of hierarchical and non-hierarchical approaches [30, 31]. In brief, first, Ward’s method based on squared Euclidean distances was applied as a hierarchical cluster analysis, and several possible cluster solutions were identified and compared. Second, a non-hierarchical K-means cluster algorithm was applied with a predefined maximum 100 iterations to further refine the preliminary solution by optimizing the classification. Finally, the stability of the cluster solution was examined by randomly splitting the database into halves and repeating the same clustering procedure and comparing the solution stability (the kappa degree of concordance values for the first and second halves were 0.877 and 0.942, respectively). As a result of cluster analysis, a new categorical variable was generated, with as many categories as the number of clusters derived. Since clusters are mutually exclusive and each category corresponds to a cluster, participants are therefore included in a single category.

Statistical differences were identified by chi-square test for categorical variables and by analysis of variance (one-way ANOVA) for continuous variables and Bonferroni correction was used to adjust for multiple pairwise comparisons. Linear mixed-effect models were used to assess the association between lifestyle clustering and anthropometric indicators. Fixed effects were fasting status (yes/no), maturation stage (continuous, scale 1 to 5/unknow), migrant background (yes/no/unknow), and parental educational level (low/average/high/unknow). Region (Madrid or Barcelona) and schools were handled as random effects.

The associations between lifestyle patterns and overweight/obesity (z-BMI percentile ≥ 85^th^) or central obesity (z-WC percentile ≥ 90^th^) prevalence were assessed with generalized mixed models using a Poisson distribution with a log link and robust error variance. In these cases, the same fixed effects and random effects described above were applied.

Statistical significance was set at a threshold of p < 0.05. Dietary and lifestyle patterns derived by principal component analysis and cluster analysis, respectively, as well as the descriptive statistics, were carried out with SPSS Statistics version 23.0 (Armonk, NY: IBM Corp). Generalized mixed models were carried out using Stata version 15 (StataCorp, College Station, Texas).

## Results

The analysis included a total of 1183 adolescents aged 12.5 (0.4) years (50.5% girls) from schools located in Barcelona (*n* = 818) and Madrid (*n* = 365).

**Fig. 1:**
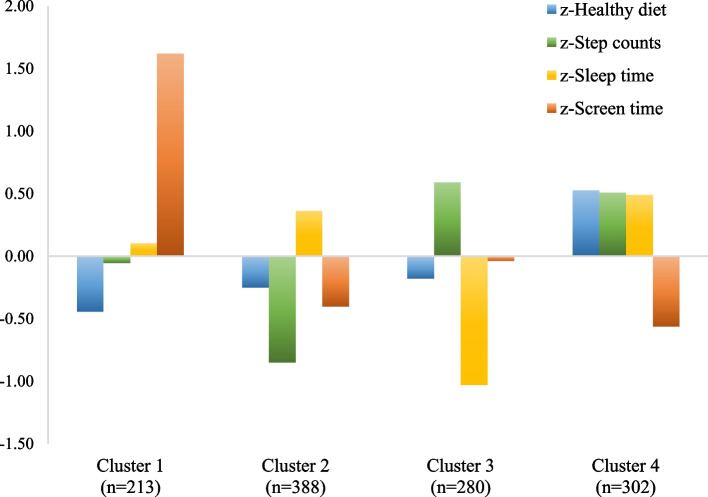
Final cluster centres. Mean z-scores for lifestyle behaviors (Healthy diet component, step counts, sleep time, and screen time) in the four clusters identified in adolescent participants of the SI! Program

Four lifestyle clusters were identified (Fig. 1 and Table 1). Participants in *Cluster 1 (C1)* (*n* = 213, 46.9% girls) showed the highest leisure screen time scores and the lowest scores in healthy diet. Participants in *Cluster 2 (C2)* (*n* = 388, 60.3% girls) only scored above average in sleep time and presented the lowest step counts. Participants in *Cluster 3 (C3)* (*n* = 280, 41.8% girls) presented the lowest sleep time score and the highest step counts score. Finally, *Cluster 4 (C4)* (*n* = 302, 48.3% girls) showed the healthiest lifestyle profile with scores above average in healthy diet, step counts and sleep time, and the lowest score in leisure screen time.

**Table 1:** Participant characteristics by cluster

	**Overall (*n* = 1183)**	**Cluster 1 (*n* = 213)**	**Cluster 2 (*n* = 388)**	**Cluster 3 (*n* = 280)**	**Cluster 4 (*n* = 302)**	***p*-value**
**Age, y**	12.5 (0.4)	12.6 (0.5)^a^	12.5 (0.4)	12.5 (0.4)	12.5 (0.4)^a^	**0.016**
**Gender, % girls**	597 (50.5%)	100 (46.9%)	234 (60.3%)	117 (41.8%)	146 (48.3%)	** < 0.001**
**Parental origin**						
Spanish	799 (67.5%)	143 (67.1%)	273 (70.4%)	179 (63.9%)	204 (67.5%)	0.409
Migrant background	371 (31.4%)	68 (31.9%)	112 (28.9%)	95 (33.9%)	96 (31.8%)	
Unknown	13 (1.1%)	2 (0.9%)	3 (0.8%)	6 (2.1%)	2 (0.7%)	
**Parental education level**						
Low	213 (18.0%)	63 (29.6%)	55 (14.2%)	36 (12.9%)	59 (19.5%)	** < 0.001**
Medium	484 (40.9%)	97 (45.5%)	159 (41.0%)	131 (46.8%)	97 (32.1%)	
High	474 (40.1%)	51 (23.9%)	172 (44.3%)	108 (38.6%)	143 (47.4%)	
Unknown	12 (1.0%)	2 (0.9%)	2 (0.5%)	5 (1.8%)	3 (1.0%)	
**Household income**						
Low	381 (32.2%)	89 (41.8%)	116 (29.9%)	90 (32.1%)	86 (28.5%)	** < 0.001**
Medium	367 (31.0%)	74 (34.7%)	121 (31.2%)	96 (34.3%)	76 (25.2%)	
High	420 (35.5%)	49 (23.0%)	148 (38.1%)	88 (31.4%)	135 (44.7%)	
Unknown	15 (1.3%)	1 (0.5%)	3 (0.8%)	6 (2.1%)	5 (1.7%)	
**Lifestyle variables**						
Vegetables, times/week	7.8 (7.5)	5.5 (5.7)^a^	6.6 (5.4)^b^	6.9 (6.1)^c^	11.9 (10.2)^a,b,c^	** < 0.001**
Fruit, times/week	7.5 (7.3)	5.7 (6.7)^a^	6.5 (6.4)^b^	6.9 (6.7)^c^	10.4 (8.3)^a,b,c^	** < 0.001**
Fast food, times/week	2.7 (3.6)	3.6 (4.4)^a,d,e^	2.5 (3.2)^d^	2.7 (3.4)^e^	2.4 (3.6)^a^	** < 0.001**
Sweetened drinks, times/week	4.5 (7.4)	7.3 (10.6)^a,d,e^	4.0 (6.5)^d^	3.9 (6.9)^e^	3.6 (5.7)^a^	** < 0.001**
Sweets, times/week	8.9 (9.8)	11.7 (12.4)^a,d,e^	8.2 (7.8)^d^	8.5 (10.6)^e^	8.0 (9.1)^a^	** < 0.001**
Step counts/day, × 1000	12.0 (2.4)	11.9 (2.1)^a,d,e^	10.0 (1.4)^b,d,f^	13.5 (2.0)^e,f^	13.3 (1.9)^a,b^	** < 0.001**
Sleep time, h/day	7.7 (0.8)	7.8 (0.7)^a,d,e^	8.0 (0.6)^d,f^	6.8 (0.6)^c,e,f^	8.1 (0.6)^a,c^	** < 0.001**
Screen time, h/day	3.9 (1.5)	6.3 (1.0)^a,d,e^	3.3 (0.9)^b,d,f^	3.8 (1.0)^c,e,f^	3.0 (0.9)^a,b,c^	** < 0.001**
**Nutritional status**						
Underweight	35 (3.0%)	11 (5.2%)	12 (3.1%)	5 (1.8%)	7 (2.3%)	**0.011**
Normal weight	830 (70.2%)	139 (65.6%)	279 (71.9%)	187 (66.8%)	225 (74.5%)	
Overweight	204 (17.3%)	34 (16.0%)	73 (18.8%)	51 (18.2%)	46 (15.2%)	
Obesity	113 (9.6%)	28 (13.2%)	24 (6.2%)	37 (13.2%)	24 (7.9%)	

*Cluster 1-higher screen time and poorer diet; Cluster 2-lower activity and longer sleepers; Cluster 3-active and shorter sleepers; and Cluster 4-healthiest*

Values are expressed as mean (standard deviation) for continuous variables or as frequency (percentage) for categorical variables. p-values for cluster differences were calculated by ANOVA or chi-square test, as appropriate. Significant differences between clusters after Bonferroni correction (*p *< 0.05) are presented in bold: a, *Cluster 1* vs *Cluster 4*; b, *Cluster 2* vs *Cluster 4*; c, *Cluster 3* vs *Cluster 4*; d, *Cluster 1* vs *Cluster 2*; e, *Cluster 1* vs *Cluster 3*; f, *Cluster 2* vs *Cluster 3*. Migrant background was assumed when at least one of the parents was born outside Spain. Parental education level was categorized according to the International Standard Classification of Education (ISCED). Household income was categorized based on the average annual household income in Spain in 2016 (26,730 €). Nutritional status was defined according to the Centers for Disease Control (CDC) standards

The clusters differed in all the evaluated lifestyle variables and in the socioeconomic characteristics, except in the migrant background (Table 1). Participants in *C1* included the highest percentage of families with lower education (29.6%) and income (41.8%) levels, while *C4* included a higher percentage of families with high socioeconomic status (47.4% of high parental education and 44.7% of high household income levels). Significant differences between clusters in the nutritional status were also observed, showing *C3* the highest prevalence of overweight/obesity (31.4%) and *C4* the lowest (23.1%).

**Fig. 2:**
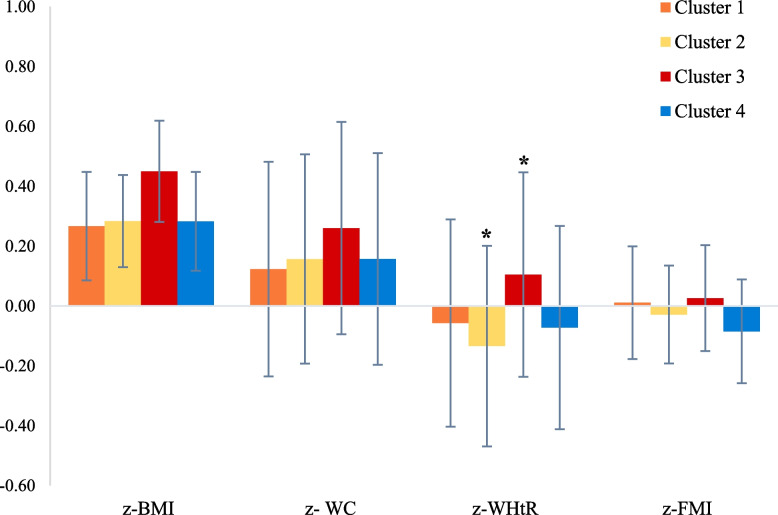
Mean z-scores and 95% confidence intervals for anthropometric variables by lifestyle behavior cluster. Estimated marginal mean z-scores (95% confidence intervals) obtained from multilevel linear mixed-effects models. Fixed-effect variables were fasting status, maturation stage, migrant background, and parental education level. Region and school were handled as random effects to account for the clustered study design. *Cluster 1-higher screen time and poorer diet*; *Cluster 2-lower activity and longer sleepers*; *Cluster 3-active and shorter sleepers*; *and Cluster 4-healthiest*. z-BMI, body mass index; z-WC, waist circumference; z-WHtR, waist-to-height ratio; z-FMI, fat mass index. Significant differences between clusters after Bonferroni correction (*p* < 0.05): * *Cluster 2 vs Cluster 3*

The associations of lifestyle clusters with anthropometric indicators are shown in Fig. 2. Participants in *C3* had the least favorable overall anthropometric profile, although significant differences were only found in z-WHtR against *C2*.

Adjusted models show that adolescents in *C1, C2,* and *C3* showed a higher prevalence of overweight/obesity than the cluster with the healthiest lifestyle profile (*C4*): *C1* PR: 1.12 (95% CI: 1.11, 1.13); *C2* PR: 1.07 (95% CI: 1.02, 1.12); and *C3* PR: 1.31 (95% CI: 1.31,1.31) (Table 2). *C3* also showed a higher prevalence of central obesity than *C4* (PR: 1.40 [95% CI: 1.33, 1.47]).

**Table 2:** Prevalence ratio of overweight/obesity and central obesity by cluster

	Model 1*		Model 2*	
	**Overweight/obesity (*n*=1182)**	**Central obesity (*n*=1183)**	**Overweight/obesity (*n*=1132)**	**Central obesity (*n*=1133)**
**Cluster 1**	**1.26 (1.10, 1.45)**	1.27 (0.81, 1.98)	**1.12 (1.11, 1.13)**	1.16 (0.92, 1.45)
**Cluster 2**	**1.15 (1.03, 1.28)**	**0.88 (0.81, 0.95)**	**1.07 (1.02, 1.12)**	**0.73 (0.69, 0.76)**
**Cluster 3**	**1.35 (1.27, 1.42)**	**1.47 (1.45, 1.50)**	**1.31 (1.31, 1.31)**	**1.40 (1.33, 1.47)**
**Cluster 4**	[Reference]	[Reference]	[Reference]	[Reference]

*Cluster 1-higher screen time and poorer diet; Cluster 2-lower activity and longer sleepers; Cluster 3-active and shorter sleepers; and Cluster 4-healthiest*. Overweight and obesity defined as age- and sex-adjusted body mass index percentiles were 85^th^-95^th^ and > 95^th^, respectively, according to the Centers for Disease Control (CDC) standards. Central obesity was defined by 90^th^ percentile according to the Third National Health and Nutrition Examination Survey (NHANES III)

^*^ Prevalence ratio from generalized models using a Poisson distribution with a log link and robust error variance. Model 1: Region and school were handled as random effects to account for the clustered study design. Model 2 was additionally adjusted for fasting status, maturation stage, migrant background, and parental education level were included as fixed-effect variables. Significant differences (*p* < 0.05) are presented in bold

## Discussion

In a large sample of early adolescents included in the SI! Program for Secondary Schools in Spain, four lifestyle clusters were derived from four key lifestyle behaviors (healthy diet, step counts, sleep time, and leisure screen time). Three of them (*C1*, *C2*, and *C3*) showed mixed behavioral profiles, while the *C4* was nearest to a healthy profile. Except for the number of steps, which was above the recommendations, the other lifestyle behaviors described in the clusters presented levels far below recommendations. The clusters showed an unequal distribution of the participants with respect to the socioeconomic characteristics. Adolescents in *C3-active and shorter sleepers* had the most unfavorable anthropometric profile and the highest prevalence of overweight/obesity or central obesity; in contrast adolescents in *C4-healthiest* showed the most favorable body composition profiles. These findings are relevant and suggest that adolescents with relatively shorter sleep time were at particular high risk of presenting an unhealthier adiposity profile, even if they presented high activity levels.

Socioeconomic differences during childhood and adolescence are relevant, since their effect in lifestyle accumulates over time, broadening the gap in health inequalities.

Previous studies reported that adolescents with high parental education level are often allocated to healthier clusters (better diet quality, lower sedentary behavior, or higher physical activity levels), while adolescents with low parental education level are frequently allocated to less favorable clusters [7, 32]. Socioeconomic differences were confirmed in our study, with the healthier cluster having the largest proportion of high parental education and high household income levels.

The relationship between childhood and adolescent lifestyle patterns and health indicators has been widely described, although no consistent trends were found [6, 13, 30–36]. The literature shows that lifestyles play an important role in CV health [6, 13, 33, 35–38]. Our results showed that participants in *C4-healthiest* presented the most desirable lifestyle and the most favorable anthropometric profile. In contrast, *C3-active and shorter sleepers* presented the worst anthropometric profile. Regarding nutritional status, participants in all clusters, and mainly in *C3*, showed a higher probability of having central obesity and/or overweight/obesity than the participants in the healthiest cluster (*C4*). One of the possible explanations for the higher prevalence of overweight/obesity in *C3* compared to *C4,* might be sleep time, due to the relatively large differences between these clusters. The association between short sleep duration and adverse CV health has been widely described in the literature [10, 30, 39, 40].

It is important to note that the results of cluster analysis are intrinsically related to the study sample and are limited by its behavioral levels, representing participants with better or worse healthy lifestyle profiles. In this sense, it is noteworthy that our sample presents unhealthy lifestyle habits as a whole, similarly to previous results related to lifestyle recommendations [35]. In our sample, a low percentage of adolescents met the recommendations for sleep (9-11 h/day: 4.8%), screen time (≤ 2 h/day: 6.3%), and fruit and vegetable intake (≥ 5 portions/day: 7.9%) [41–43], while physical activity was the only behavior showing predominantly adequate levels of performance with 78.9% of the sample accumulating ≥ 10,000 step counts/day [44]. Therefore, it is important to stress that clusters with the best scores for the selected behaviors do not necessarily reflect that the adolescents included therein met the recommendations (e.g., only 19.5% and 12.9% of participants in *C4* met the fruit and vegetable consumption and screen time recommendations, respectively). Yet, the cluster solution was able to detect specific groups of adolescents at particular higher risk of presenting overweight/obesity according to four important lifestyle behaviors.

This study has some limitations. This cross-sectional analysis allows the establishment of associations, but not causation. Participation was voluntary, and we therefore cannot exclude selection bias and adolescents with worse CV health or unhealthier lifestyles might have been less willing to participate. In this analysis, only participants with complete information on the behaviors involved in the derived cluster solution were included, showing the excluded participants a differential sociodemographic distribution and thus resulting in a potential additional bias (Additional file 1). Another limitation is that early adolescents engaged in sports were often asked by coaches to remove their accelerometers during training and competition, resulting in potentially underestimated physical activity and therefore we decided to report step counts as an overall indicator of physical activity. Finally, the use of questionnaires introduces the possibility of social desirability bias and unreliability of estimated food intake frequencies or daily leisure screen time.

Strengths of the study include the status of the SI! Program for Secondary Schools as a large, longitudinal multi-center study conducted in early adolescents who were assessed through a solid combination of direct measurements and questionnaires, all carried out by trained nutritionists and nurses. Another strength is the use of wrist-worn accelerometers [21] that provide better sleep estimates for adolescents compared to waist-worn accelerometry data [45].

## Conclusions

Family sociodemographic determinants were linked to the distribution of the four lifestyle clusters identified. Participants in the healthiest lifestyle pattern profile showed the lowest prevalence of overweight/obesity, while participants in the cluster with the relatively lowest sleep time showed the highest prevalence of overweight/obesity and central obesity. Nevertheless, except for the number of steps, the lifestyle behaviors described in the four clusters presented levels far from the recommended values; thus, finding differences between clusters that reflect the importance of complying with the overall lifestyle recommendations was not feasible. Follow-up of this study cohort will allow to assess the evolution of behaviors that appear to influence CV health in adolescents and the potential effects of a health promotion program.

### Supplementary Information

**Additional file 1.**

## Data Availability

Data availability to external researchers is restricted to related project proposals upon request to the corresponding author. Based on these premises, deidentified participant data will be available with publication after approval of the proposal by the Steering committee and a signed data sharing agreement.

## Abbreviations

BMI: Body mass index
C1: Cluster 1
C2: Cluster 2
C3: Cluster 3
C4: Cluster 4
CDC: Center for Disease Control
CV: Cardiovascular
FMI: Fat mass index
ISCED: International Standard Classification of Education
NHANES III: Third National Health and Nutrition Examination Survey
SI!: *Salud Integral*
WC: Waist circumference
WHtR: Waist-to-height ratio
