# Supplementary material for: Clustering of lifestyle behaviors and adiposity in early adolescents in Spain: findings from the SI! Program for Secondary Schools
Source: BMC Public Health. 2023 Aug 12;23:1535. doi: 10.1186/s12889-023-16461-6 (PMC10422708; doi:10.1186/s12889-023-16461-6)
Supplement: Supplementary file 1 — Additional file 1. [file 12889_2023_16461_MOESM1_ESM.docx]

**SUPPLEMENTARY INFORMATION:**

**Additional file 1:**

- **File name**: 2022.11.25_Additional file 1_Lifestyle cluster and adiposity_BMCPublicHealth.docs
- **Title of data**: Description of the socioeconomic variables of the participants included vs excluded from the analysis
- **Description of data**: sensitivity analysis comparing participants included vs excluded from the analysis in terms of basic sociodemographic variables

**Additional file 1** Description of the socioeconomic variables of the participants included vs excluded from the analysis.

|  | **Included (n=1183)** | **Excluded**  **(n=143)** | **p-value** |
| --- | --- | --- | --- |
| **Age, y** | 12.5 (0.4) | 12.7 (0.6) | **<0.001** |
| **Gender, % girls** | 597 (50.5%) | 45 (31.5%) | **<0.001** |
| **Parental origin** |  |  |  |
| Spanish | 799 (67.5%) | 79 (55.2%) | **0.001** |
| Migrant background | 371 (31.4%) | 58 (40.6%) |  |
| Unknown | 13 (1.1%) | 6 (4.2%) |  |
| **Parental education level** |  |  |  |
| Low | 213 (18.0%) | 33 (23.1%) | **0.032** |
| Medium | 484 (40.9%) | 53 (37.1%) |  |
| High | 474 (40.1%) | 52 (36.4%) |  |
| Unknown | 12 (1.0%) | 5 (3.5%) |  |
| **Household income** |  |  |  |
| Low | 381 (32.2%) | 55 (38.5%) | **0.012** |
| Medium | 367 (31.0%) | 43 (30.1%) |  |
| High | 420 (35.5%) | 39 (27.3%) |  |
| Unknown | 15 (1.3%) | 79 (55.2%) |  |

Values are expressed as mean (standard deviation) for continuous variables or as frequency (percentage) for categorical variables. p-values for cluster differences were calculated by ANOVA or chi-square test, as appropriate. Significant differences (p<0.05) are presented in bold. Migrant background was assumed when at least one of the parents was born outside Spain. Parental education level was categorized according to the International Standard Classification of Education (ISCED). Household income was categorized based on the average annual household income in Spain in 2016 (26730 €).
